# Comparison of intubation and tracheotomy in adult patients with acute epiglottitis or supraglottitis

**DOI:** 10.1007/s00405-019-05624-0

**Published:** 2019-09-05

**Authors:** Laura K. Tapiovaara, Katri L. S. Aro, Leif J. J. Bäck, Anni I. M. Koskinen

**Affiliations:** Department of Otorhinolaryngology, Head and Neck Surgery, Helsinki University Hospital and University of Helsinki, PO Box 263, 00029 HUS Helsinki, Finland

**Keywords:** Airway, Head and neck, Infection, Cost, Complication

## Abstract

**Purpose:**

In acute epiglottitis (AE) or acute supraglottitis (AS), the management of the airway is crucial. We hypothesized that tracheotomized patients recover faster than intubated patients do.

**Methods:**

We retrospectively reviewed all adult AE and AS patients, who underwent intubation or tracheotomy between 2007 and 2018 in a tertiary care center. Patient demographics, treatment, and complications were analyzed.

**Results:**

The cohort comprised 42 patients. The airway was secured with intubation in 50% and with tracheotomy in 50%. All intubated patients (*n* = 21) and three tracheotomized patients were treated in the intensive care unit (*p* < 0.0001). Procedure-related complications were encountered in three intubated and eight tracheotomized patients (*p* = 0.892). Median overall treatment cost was 11.547 € and 5.856 € in the intubated and tracheotomized patient groups, respectively (*p* < 0.001). The median duration of sick leave after discharge from hospital was 13 days in the tracheotomy group and 7 days in the intubation group (*p* = 0.097).

**Conclusion:**

Tracheotomy resulted in a less expensive management in securing the airway in AE or AS, but tracheotomized patients had a trend towards more complications and longer sick leaves compared to intubated patients.

**Level of evidence:**

2b

## Introduction

Acute epiglottitis (AE), or supraglottitis (AS), is a potentially life-threating condition, in which the inflammation and edema of the epiglottis and/or adjacent supraglottic structures may endanger the airway within hours [1]. In the post *Hemofilus influenzae* type b vaccine era, there has been a shift in AE and AS from the stereotypical children’s disease towards affecting the adult population [2, 3]. In fact, in Finland the incidence of AS in adults has increased from 1.88 in the 1990sss to 4.73 in the twenty-first century per 100,000 inhabitants [4].

Management of the airway is crucial in a severe AE/AS, but an airway procedure is required in under 10% of the cases [4–6]. The presence of stridor, tachypnea, hypoxia, or drooling at the time of diagnosis indicates the need for an airway procedure [4]. In addition, older age, male sex, BMI over 25, and diabetes mellitus increase the risk for the necessity for an airway intervention [5]. However, it is still unknown which airway management method is preferred. The aim of the present study was to evaluate the management of the airway by intubation or tracheotomy in adult patients with AE or AS. We hypothesized that favoring tracheotomy in such cases would enable patients to recover faster.

## Materials and methods

This retrospective study was conducted in the Helsinki University Hospital, Helsinki, Finland with a referral area of 1.6 million people. Patient data between May 1, 2007 and November 30, 2018 were retrieved from the electronic operative files. We included all adult (18 years and older) patients who were treated at the operative theatre at the Helsinki University Hospital with the diagnosis code (ICD-10) of J05.0 (which stands for AS) or J05.1 (which stands for AE). We included only procedures coded for intubation (WX704 and WX707) or tracheotomy (GBB00 and GBA00). Patients with uncertain diagnoses and other simultaneous procedures were excluded from further analysis. This study was based on hospital and patient records without any ethical issues and an institutional research permission was granted (HUS/66/2018, October 9, 2018).

Patient demographics, the onset of symptoms, management of the airway, timing of extubation and decannulation, bacterial findings, chest X-ray findings, highest CRP level while hospitalized, length of stay in the intensive care unit (ICU) and hospital ward, length of sick leave starting from the day of discharge from the hospital until the patient returned to work (for patients in working life), 30-day postoperative complications, and follow-up findings were manually registered from the electronic hospital charts and transferred to SPSS statistical software by two authors (LT and AK). Patient comorbidities were retrospectively evaluated from the hospital charts using adult comorbidity evaluation ACE-27 [7]. The severity of postoperative complications was assessed retrospectively according to Clavien–Dindo (CD) classification [8].

Overall cost was calculated multiplying daily cost of care by treatment days in the ICU or ward. In 2018, a day in the ICU in the Helsinki University Hospital cost 3.117 € and a day in the ward 732 €, including all necessities required during care.

We used descriptive statistics to summarize frequencies, proportions, medians, and ranges. Pearson’s Chi-square test with asymptotic and exact *p* values was used when best appropriate to calculate the statistical differences between categorical variables, and independent-samples *t* test was used for continuous variables. A two-sided *p* value of less than 0.05 was considered statistically significant. SPSS version 24 (SPSS, Inc. Chicago, IL, USA) served in all statistical analyses.

## Results

The airway was secured with either intubation or tracheotomy in 63 adult patients with AE or AS. From these, 17 were excluded because of non-specific diagnosis, and 4 because of another simultaneous procedure. Thus, 42 patients (27 AE, 15 AS) formed the study cohort, and we divided them into two subgroups based on airway management. One patient was lost to follow-up. Two patients required an emergency cricothyroidotomy with tracheotomy immediately thereafter, and they were included in the tracheotomy group. Three patients were initially intubated, but tracheotomy was performed later (median 6 days; range 3–8), and they were included in the intubation group.

The baseline demographics, median duration of total hospital and ICU stay, length of sick leave, and overall cost of care are presented in Tables 1and 2. There were no statistically significant differences between groups in terms of sex, age, comorbidities, or smoking (Table 1). In 21 patients (50%), the airway was managed with intubation, and in 21 patients (50%) with tracheotomy. In AE, tracheotomy was the selected method in 44% of patients, and in AS in 60% of patients (*p* = 0.340; Table 1). All tracheotomized patients were decannulated at the end of the study period, in the median of 5 days (range 3–27).

**Table 1:** Baseline demographics, duration of antibiotic treatment and highest plasma CRP (mg/ml) level

Characteristics	All patients (%) (*n* = 42)	Tracheotomy (%) (*n* = 21)	Intubation (%) (*n* = 21)	*p* value
Sex				1.000
Male	32 (76)	16 (38)	16 (38)	
Female	10 (24)	5 (12)	5 (12)	
Diagnosis				0.340
Acute epiglottitis	27 (64)	12 (29)	15 (36)	
Supraglottitis	15 (36)	9 (21)	6 (14)	
Median age, years (range)	49 (17–80)	51 (31–80)	45 (17–67)	0.345
Comorbidity ACE-27				0.549
0	21 (50)	11 (26)	10 (24)	
1	10 (24)	6 (14)	4 (10)	
2	9 (21)	3 (7)	6 (14)	
3	2 (5)	1 (2)	1 (2)	
Smoking				0.273
No	8 (19)	5 (12)	3 (7)	
Yes	16 (38)	7 (17)	9 (21)	
Median duration of iv-antibiotics; days (range)	7 (3–27)	7 (4–27)	7 (3–16)	0.656
Median of the highest plasma CRP level; (range)	207 (45–483)	228 (104–362)	180 (45–483)	0.204

*iv* intravenous, *CRP* C-reactive protein

**Table 2:** Duration of symptoms, length of total hospital stay, length of ICU care, length of sick leave, intubation and tracheotomy period, and overall cost

Median duration (days; range)	All patients	Tracheotomy	Intubation	*p* value
Symptoms before admittance to hospital	2 (1–7)	2 (1–7)	3 (1–7)	0.118
Hospital stay	6 (3–25)	8 (5–25)	6 (3–25)	0.274
Patients treated in the ICU (*n*)	24	3	21	0.0001*
ICU care	3.5 (1–16)	5 (3–5)	3 (1–16)	0.683
Sick leave	7 (3–23)	13 (3–23)	7 (3–14)	0.097
Median overall cost € (range)	10.083 (3.660–56.460)	5.856 (3.660–21.063)	11.547 (5.313–56.460)	0.0001*
Intubation period			3 (1–14)	
Tracheotomy period	5 (3–27)	5 (3–27)	6 (3–8)^b^	0.894

*ICU* intensive care unit

*Statistically significant

^b^3 patients were initially intubated

All patients in the intubation group and three patients in the tracheotomy group were treated in the ICU (*p* < 0.001). The three tracheotomized patients treated in the ICU needed ventilation support and one of them received treatment also for delirium. No difference appeared in overall hospital stay or duration of ICU care between the different airway management groups (Table 2). Median overall cost was 11.547 € in the intubation group and 5.856 € in the tracheotomy group (*p* < 0.001; Table 2.). The median duration of sick leave after discharge from hospital was 13 days in the tracheotomy group and 7 days in the intubation group; however, the difference was not significant.

The median of the highest plasma CRP level was 228 mg/l (range 104–362) in the tracheotomy group, and 180 mg/l (range 45–483) in the intubation group (*p* = 0.204). Blood culture was positive in three patients in the tracheotomy and in 1 patient in the intubation group: *Streptococcus pneumoniae* (*n* = 2), *Neisseria meningitidis* (*n* = 1), and *Streptococcus pyogenes* (*n* = 1). Five patients in the tracheotomy group and four patients in the intubation group had pneumonia according to their chest X-ray.

## Complications

Altogether, 13 complications were recorded; in 8 patients in the tracheotomy group, and in 3 patients in the intubation group (*p* = 0.892). Most of them were classified as CD class I–II. Five patients (22%) in the tracheotomy group had stoma necrosis or an infection around the stoma (CD class II). Unilateral recurrent laryngeal nerve paresis occurred in 1 patient (4%) in the tracheotomy group (CD class I), with full recovery after 8 months. One patient (4%), who required an emergency cricothyroidotomy and was treated in the ICU had glossopharyngeal nerve paresis (CD class I) and decubitus ulcers in the lower back (CD class I). The nerve paresis recovered in 5 months and was suspected to be induced by the infection. One patient (4%) in the tracheotomy group developed an ileus (CD class IIIa) needing an invasive procedure, and delirium (CD class IVa) requiring care in the ICU. Two patients (11%) in the intubation group developed vocal cord granulomas (CD class I), which resolved in 6 and 17 months. One intubated patient (5%) showed elevated cardiac troponin levels after early stage hypoxemia (CD class II), which was managed with medication and did not cause any long-term symptoms. Persistent tracheocutaneous fistulas were not encountered, and none of the fistulas required surgical intervention.

## Discussion

In the present study, we evaluated management of the airway in adult patients with severe AE or AS. Complications occurred in eight tracheotomized patients and in three intubated patients. The two encountered nerve paresis in the tracheotomy group resolved in follow-up and were thus considered to result from the infection, not the procedure itself. One tracheotomized alcoholic patient developed severe complications (ileus and delirium). We report no differences between the two groups based on their management of the airway and in terms of patient demographics. However, all intubated patients required ICU care, and their overall cost of treatment was significantly higher. Intubating a patient with AE or AS requires an experienced anesthesiologist, a well-prepared schema, and unambiguous communication between the team as intubation may fail resulting in loss of airway. We hypothesized that the tracheotomized patients recover faster, but the results from the present study do not clearly support this hypothesis, but need further assessment in larger cohorts. Nevertheless, we would recommend tracheotomy in AE or AS, since these patients require treatment in the ICU only infrequently, which inevitably decreases the cost of treatment. However, patient factors, especially comorbidities, and experience of the surgical team should be taken into consideration while choosing the optimal method for airway management.

At present, there are no conclusive data of the superiority of tracheotomy versus intubation in the management of the airway in AE or AS. Intubation is regarded as easy and rapid, associated with low cost and avoidance of acute and late surgical complications [9]. The possible advantages after tracheotomy may include less need for sedation, better patient comfort, and the possibility to communicate and swallow [9]. Often, the method of airway management must be assessed on case-by-case basis. We had slightly more tracheotomies in the AS group compared to the AE group (60% and 44%, respectively). In AS, the edema of the supraglottic area is multilocular, compared to AE, where only the epiglottis is swollen. The larger area affected by the profound edema may favor the use of tracheotomy. In cases of AE or AS with near-total obstruction of the airway, intubation may not even be an option. We experienced two such cases, both had AE, where emergency cricothyroidotomy was the only possibility to secure the airway.

In the present study, most patients with tracheotomy were treated in a hospital ward only. They required no sedation and were able to move freely, communicate, and eat and drink almost immediately after hospitalization. However, three tracheotomized patients needed treatment in the ICU because of ventilation deficiency; all of them had synchronous pneumonia. One of them also had seizures and developed delirium due to previous alcohol abuse. On the contrary, all intubated patients were treated in the ICU, and were thus forced to stay in bed. Bed rest has potential complications, for example pulmonary edema, atelectasis, muscle wasting, and the risk for blood clots [10]. Furthermore, it is impossible to evaluate possible dysphagia, and rehabilitate swallowing in intubated patients.

Patients in the tracheotomy group had longer sick leaves, although the difference was insignificant. The doctor who discharged the patient from hospital assessed the required length of sick leave. It is possible that the remaining open stoma was the reason for longer sick leaves although our experience is that the closure is nearly complete within a week after such a short cannulation period. This was, however, not evaluated in this study. In head and neck cancer patients, early suturing after decannulation seems effective, as it promotes commencement of swallowing and earlier discharge from the hospital [11]. Another study on head and neck cancer patients shows early surgical closure of tracheostoma to be simple and safe, with minimal postoperative morbidity [12]. We believe the same would apply in patients with AE or AS and recommend that immediate surgical closure of the stoma after decannulation is worth considering to precipitate recovery.

Since all intubated patients were treated in the ICU, the treatment cost was almost two times higher than those of tracheotomized patients who were mainly treated in a hospital ward. To our knowledge, this is the first study to report cost analysis among intubated or tracheotomized AE or AS patients. Hyde et al. showed that among trauma patients, early tracheotomy is less expensive than late tracheotomy because it decreases morbidity and length of ICU care [13]. At our institution, the cost of ICU care is over four times higher than the cost of regular ward care per day. In addition, avoiding care in the ICU whenever possible might increase additional savings when limiting possible complications as discussed previously.

We did not encounter any significant differences in complication rates, although more patients in the tracheotomy group developed a complication, and these were commonly infections around the stoma. Bontempo and Manning report an overall complication rate of tracheotomy covering all indications to be up to 50%, but most of them were regarded as minor complications [14]. Of note, not all studies regard stoma infection as a complication [15]. A recent paper studied the effect of antibiotic prophylaxis with clindamycin versus placebo (600 mg every 8 h for 24 h vs. saline) in surgical tracheotomies and found the infection rate to drop from 23 to 7% [16]. Since all patients in the present study had AE or AS, they received intravenous antibiotics preoperatively, which continued for several days thereafter. The late complications after airway management in AE or AS need evaluation in future studies, as known late complications of tracheotomy and intubation include for example tracheal stenosis [17, 18] and tracheocutaneous fistula after tracheotomy [19], and laryngeal granulomas and arytenoid cartilage dislocation after intubation [20, 21]. Our recent study reported surgical mortality to be 0.4% in all tracheotomized patients (*n* = 255) in our department, with AE or AS comprising only seven of indications for the procedure [22].

Our study includes some limitations. Due to the retrospective study design, we did not have routine follow-up visits, which would have possibly revealed some additional late complications following intubation and tracheotomy. Furthermore, we do not have data on the timing of spontaneous closure of the stoma. In addition, the cost calculations are directional. All financial elements included in care are difficult to assess retrospectively. Due to the lack of consensus on the management of the airway in AE or AS, the judgment of the preferred method in this study was often left to the preference of the otorhinolaryngologist in charge. We present the first study, to our knowledge, to compare airway management in adult population with AE or AS.

## Conclusion

Tracheotomy proved to be safe, although long-term sequelae of tracheotomy and intubation require follow-up data to objectively analyze procedure-related complications. We found major differences in the cost of care while managing patients with AE or AS. Tracheotomy seemed as a more economical way to manage the airway. Even though we did not assess the benefit of closure of the stoma after decannulation, we feel that this method would diminish the need for sick leave even further decreasing the total cost.
